# Supplementary material for: Resolving the full spectrum of human genome variation using Linked-Reads
Source: Genome Res. 2019 Apr;29(4):635–45. doi: 10.1101/gr.234443.118 (PMC6442396; doi:10.1101/gr.234443.118)
Supplement: Supplemental Material [file supp_gr.234443.118_Supplemental_longranger-2.2.zip › longranger-2.2.tar.bz2!/lr2.2-public/lib/go/src/code.google.com/vitess/vitess.io/_layouts/base.liquid]

{{ site.title }} / {{ page.title }}
{% include styles.liquid %}


{% if site.analytics.id %}
{% include analytics.liquid %}
{% endif %}

{% if page.tocDepth %}
{% else %}
{% endif %}
{% include nav.html %}
{{ content }}

{% include footer.html %}
{% include end-of-page-styles.liquid %}
